# Robotic partial nephrectomy after pazopanib treatment in a solitary kidney with segmental vein thrombosis

**DOI:** 10.1590/S1677-5538.IBJU.2018.0240

**Published:** 2019-09-02

**Authors:** Juan D. Garisto, Julien Dagenais, Daniel Sagalovich, Riccardo Bertolo, Brian Rini, Jihad Kaouk

**Affiliations:** 1 Glickman Urological & Kidney Institute, Cleveland Clinic, Cleveland OH, USA

## Abstract

**Objective:**

To demonstrate our surgical technique of robotic partial nephrectomy (RPN) in a patient with a solitary kidney who received neoadjuvant Pazopanib, highlighting the multidisciplinary approach.

**Materials and Methods:**

In our video, we present the case of 77-year-old male, Caucasian with 6.6cm left renal neoplasm in a solitary kidney. An initial percutaneous biopsy from the mass revealed clear cell RCC ISUP 2. After multidisciplinary tumor board meeting, Pazopanib (800mg once daily) was administered for 8 weeks with repeat imaging at completion of therapy. Post-TKI image study was compared with the pre-TKI CT using the Morphology, Attenuation, Size, and Structure criteria showing a favorable response to the treatment. Thereafter, a RPN was planned3. Perioperative surgical outcomes are presented.

**Results:**

Operative time was 224 minutes with a cold ischemia time of 53 minutes. Estimated blood loss was 800ml and the length of hospital stay was 4 days. Pathology demonstrated a specimen of 7.6cm with a tumor size of 6.5cm consistent with clear cell renal carcinoma ISUP 3 with a TNM staging pT1b Nx. Postoperative GFR was maintained at 24 ml / min compared to the preoperative value of 33ml / min.

**Conclusions:**

A multidisciplinary approach is effective for patients in whom nephron preservation is critical, providing an opportunity to select those that may benefit from TKI therapy. Pazopanib may allow for PN in a highly selective subgroup of patients who would otherwise require radical nephrectomy. Prospective data will be necessary before this strategy can be disseminated into clinical practice.

ARTICLE INFO

*Juan Garisto*

https://orcid.org/0000-0001-9530-403

Available at: http://www.intbrazjurol.com.br/video-section/20180240_Garisto_et_al

Int Braz J Urol. 2019; 45 (Video #18): -

